# Carrier testing for autosomal recessive disorders: a look at current practice in Germany

**DOI:** 10.1515/medgen-2021-2052

**Published:** 2021-05-14

**Authors:** Christian Netzer, Clara Velmans, Florian Erger, Julia Schreml

**Affiliations:** Universität zu Köln, Medizinische Fakultät und Uniklinik Köln, Institut für Humangenetik, Kerpener Straße 34, 50931Köln, Germany

**Keywords:** genetic counseling, autosomal recessive disorders, carrier testing, recurrence risk, expanded carrier screening

## Abstract

Counseling recurrence risks for monogenic disorders is one of the mainstays of human genetics. However, in practice, consultations concerning autosomal recessive disorders exceed the simple conveyance of a 25 % recurrence risk for future offspring. Medical geneticists should be aware of the multifaceted way in which autosomal recessive disorders can pose a diagnostic and counseling challenge in their daily lives and of the pitfalls they might encounter. Although the intentional or incidental detection of carrier states for autosomal recessive diseases happens more and more frequently, our current practice when clarifying their associated reproductive risks remains unsystematic and often subjectively guided. We question whether the approach of focusing on small recurrence risks for a single familial disease with extensive single-gene tests in the partner of a known carrier truly addresses the counseling needs of a couple seeking preconceptional genetic advice. Different perspectives between patients and medical practitioners (or between different medical practitioners) on “acceptable risks” or the extent to which such risks must be minimized raise the question of whether existing professional guidelines need to be clarified.

## Introduction

Autosomal recessive disorders play a prominent role in daily human genetic practice. They are found in all disease groups of clinical genetics, from purely metabolic disorders or isolated hearing loss to tumor predisposition syndromes, skeletal dysplasias or malformation syndromes with intellectual disability. The OMIM^®^ database currently lists approximately 2,600 autosomal recessive phenotypes (as of December 2020) [1].

In almost all cases, both parents of an affected individual are phenotypically unaffected heterozygous carriers for one of the causative mutations. The pathogenic variants are usually not *de novo* mutations, but have often been passed on over multiple generations within the family and can sometimes be traced back to very distant ancestors. Therefore, there are typically numerous additional carriers in the family, who often only become aware of their possible carrier state when an affected family member is diagnosed. Especially when they are in the family planning phase, they may seek genetic counseling in order to clarify the recurrence risk for their own offspring. The flow of information within families has been greatly simplified and improved by the widespread use of messenger services and other digital forms of communication. Sometimes the dismay about a seriously ill or deceased child in the family is so great that even distant relatives demand a maximum of diagnostics in the course of genetic counseling to exclude a similar disease for their own (future) children. Referring to the baseline risk of 2–3 % for congenital malformations and disorders [2] can, in our experience, only rarely resolve the fixation on the risk for this specific autosomal recessive disease that has occurred in the family, even though it is often lower by an order of magnitude.

**Table 1:**
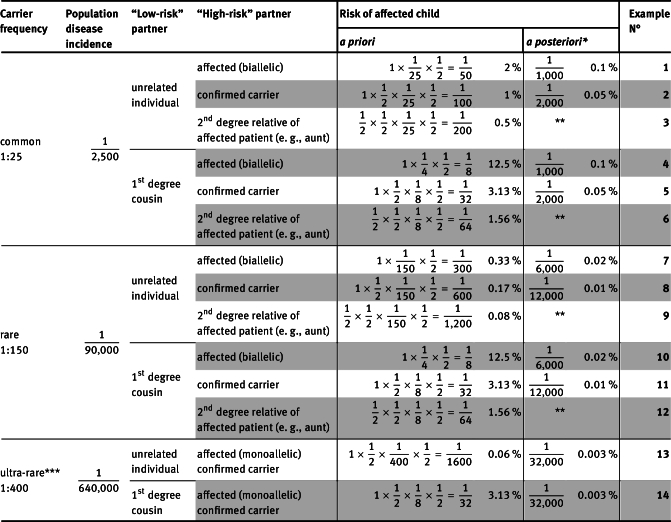
Different constellations for calculating the recurrence risk for autosomal-recessive hereditary diseases.

*: 95 % analytical sensitivity of the assay, assuming a negative result for “low-risk” partner (and in case of consanguinity: testing for the familial mutation followed by analyzing the whole gene). **: Equivalent to the scenario of the above case, if “high-risk” partner is a confirmed carrier. ***: A carrier state for an ultra-rare autosomal-recessive disorder which corresponds to an autosomal-dominant hereditary disease (e. g., *BRCA1*-associated tumor predisposition/Fanconi anemia).

## Possible constellations in genetic consultations

The textbook scenario of a genetic consultation on autosomal recessive disorders is that of a couple that already has an affected child and wishes to have further children. If both partners are molecularly confirmed carriers, the recurrence risk is 25 % (“1 in 4”). In reality, of course, there are numerous variations of this constellation, some of which are listed in Table 1. A common situation is that the mother or father of an affected child wishes to have children with a new partner, and the couple wants to rule out the possibility that their future child will also be affected by this condition. From a formal genetic perspective, the recurrence risk depends firstly on the incidence of the disease. In Table 1, we have assumed a carrier rate in the general population of 1:25 for common (e. g., cystic fibrosis in the Central European population) and 1:150 for rare autosomal recessive diseases. Another factor influencing the recurrence risk is whether there is consanguinity between the new partners; in Table 1 we have assumed a degree of relationship of first cousins for consanguineous couples. Thus, in our example the *a priori* recurrence risk can range from 1:600 (see example 8 in Table 1) to 1:32 (see examples 5 and 11). Unsurprisingly, the rarity of the disease has no bearing on the *a priori* risk in consanguineous couples.

As a first diagnostic step, a targeted carrier test for the familial mutations is performed in the partner related to the index patient (= the affected family member). If this test is positive, the other partner is offered a more comprehensive carrier test, e. g., sequencing and MLPA analysis of the corresponding gene, which is expected to detect 95 % of all mutations for our calculations in Table 1. In case no (likely) pathogenic variant is detected in the second stage of testing, the *a posteriori* risk for an affected child can be significantly reduced in each case: for a non-consanguineous couple and a common autosomal recessive disease with an *a priori* risk of 1:100, for instance, to 1:2,000 (see example 2 in Table 1). This residual risk is only slightly higher than the risk in the general population of 1:2,500. In contrast, testing for only the most frequent mutations in the partner of the confirmed carrier, with a mutation detection rate (= sensitivity) of 70 %, would only reduce the *a posteriori* risk of the couple to approx. 1:320 in this scenario (calculation not shown). Partner testing in consanguineous couples should first exclude the familial variant, after which the risk typically drops approximately to the *a priori* risk of an unrelated couple with one confirmed carrier. The risk reduction by excluding only the familial mutation may be higher in some instances, e. g., in genetically less diverse (inbred) populations or if the familial variant happens to be a more common founder variant. To truly reduce the *a posteriori* risk of consanguineous couples to the same level as the *a posteriori* risk in non-consanguineous couples, the consanguineous “low-risk” partner (as designated in Table 1) would however also have to receive comprehensive gene testing after the exclusion of the familial mutation.

With a decreasing degree of relation to the index patient, the *a priori* risk of recurrence quickly decreases as well. An affected person’s untested aunt in a non-consanguineous partnership has a 1:1,200 *a priori* recurrence risk for her offspring in the case of a rare autosomal recessive disease (see example 9 in Table 1). However, this is still a 75-fold higher risk than the risk of the general population for this disease. Sometimes, relatives are uncomfortable asking their family members for the report of genetic testing of the index patient, also because this reveals that they themselves might want to take measures to avoid a similar disease among their own offspring. The genetic report of the index patient should however clearly be sought whenever possible before a carrier test is offered. Otherwise, the costs for diagnostics might considerably increase because no targeted test for the familial mutation is possible, and additional uncertainties may arise in the test interpretation. In case of a negative test result it remains unclear whether the used method would have picked up the familial mutation.

A relatively high recurrence risk arises when a person affected by an autosomal recessive disease plans to have children. In such cases, the *a priori* risk ranges from 1:300 for rare diseases and 1:50 for common diseases in non-consanguineous couples (see examples 7 and 1 in Table 1) to 1:8 in consanguineous couples (see examples 4 and 10).

Sometimes carriership of a heterozygous variant leading to an autosomal dominant disease in a patient represents at the same time a carrier state for a – typically much more severe – autosomal recessive disorder. In these cases, the autosomal recessive phenotype will be ultra-rare, as the associated autosomal dominant disorders are already rare, and the *a priori* risk for the occurrence of the corresponding autosomal recessive phenotypes among the offspring is very low for non-consanguineous couples (<1:1,000, see example 13 in Table 1). An instance of this is the tumor predisposition syndrome HNPCC (Lynch syndrome; OMIM PS120435): biallelic mutations in the HNPCC genes lead to a *mismatch repair cancer syndrome* (OMIM PS276300), which typically manifests in early childhood. Another classic example is Fanconi anemia as a result of biallelic *BRCA1*/2 mutations (OMIM PS227650). According to a survey from the USA published in 2016, such reproductive risks are addressed by about half of the genetic counselors in the course of a diagnostic workup for suspected hereditary tumor predisposition syndromes [3].

In our institute, a relatively new constellation has emerged in recent years as a frequent reason for offering carrier testing. In the course of comprehensive gene panel analyses in children with a suspected hereditary disease, we often detect a carrier state that is probably not related to the child’s disease (see below). Such a carrier state is typically also present in (at least) one parent, and since trio analyses are not reimbursed for patients with statutory health insurance, it is initially unclear whether the other parent in each case also happens to be a carrier for the corresponding disease (but has not passed on the mutation to the child). Since the parents often have not yet completed family planning and already have a child with a congenital disease, many of them are understandably very interested in clarifying additional reproductive risks, even if such risks are objectively low. Also, the parents of an index patient are sometimes already in new partnerships at the time of genetic diagnostics for their child, so that even a trio exome analysis is unable to resolve this issue in all cases.

In the most comprehensive analyses in our institute – namely those to clarify the cause of an intellectual disability – we currently evaluate about 2,500 genes from an exome dataset [4] and find on average one (probably) pathogenic heterozygous variant in a gene that is causative for an autosomal recessive phenotype. Since the vast majority of parents explicitly consent to being informed about additional findings with relevance for family planning, we are usually obligated to report such a carrier state. The risk for a subsequent affected child in such a constellation is mostly in the range of 1:1,600 to 1:100 for the same couple, depending on the frequency of the disease (see examples 13 and 2 in Table 1), but can be much higher for consanguineous couples (1:32 for first cousins, see examples 5, 11 and 14). The simultaneous detection of a carrier state for two or even three autosomal recessive diseases in the exome-sequenced index patient is not uncommon and further increases the effort for the subsequent processing of the findings during genetic counseling of the parents. Interestingly, according to a recently published study, over half of the international laboratories surveyed do not report a carrier state in the context of trio exome analyses if they have concluded that the identified variant is not causally related to the patient’s phenotype [5]. Trio-sequencing can provide reassurance to the parents in most of these situations, since usually only one of them will be a carrier for the disease in question. Ultimately, of course, we believe this is an issue that must be discussed and decided with families beforehand as part of the informed consent process for genetic analysis.

## When does a possible carrier state need to be addressed in genetic counseling?

The current maternity guidelines (“Mutterschaftsrichtlinie”) of the German *Federal Joint Committee* (“Gemeinsamer Bundesausschuss”), passed in November 2020, state the following: If there is evidence for a genetic disease in the family, “[...] the physician is required to inform the pregnant woman about the possibilities of human genetic counseling and/or human genetic testing” [6]. According to the German *S2 Guideline for Human Genetics*, the indication for genetic counseling is for issues “related to the occurrence or increased likelihood of occurrence of an (epi)genetic [....] disease or developmental disorder” [7]. Carrier testing is explicitly mentioned at this point in the guideline. Even if the question of a carrier state only arises “incidentally” in the context of a consultation on another topic, in our opinion the medical geneticist must offer the patient a detailed discussion of this point, including an explanation of related diagnostic options. If a consultation is related to or focused on questions about reproductive genetic risks, such a detailed discussion will probably have to take place. Furthermore, point 11.1 of the guideline also demands that the possible significance of a patient’s diagnostic findings for the family planning of relatives should be explained to him/her during genetic counseling. If this procedure is implemented for autosomal recessive diseases, an increased demand for carrier testing in the sense of “cascade testing” of family members is inevitable and already a reality today.

Unfortunately, none of the abovementioned German guidelines give practical guidance on when a risk for a hereditary disease among the offspring is to be regarded as “increased” – or whether any relative increase in risk compared with the general population constitutes sufficient reason for genetic testing. It also remains an open question how comprehensive the genetic testing has to be for autosomal recessive diseases. Is the exclusion of the most frequent mutations and thus a significant reduction of the recurrence risk sufficient (for example, the exclusion of the typical heterozygous *SMN1* deletion by MLPA when there is a case of spinal muscular atrophy in the family)? If desired by the couple, do we need to escalate testing until the residual risk is equal to that of the general population? Or do we even have to continue until all currently available routine diagnostic options have been exhausted, i. e., in the abovementioned case, until also the technically challenging and time-consuming sequencing of the *SMN1* gene has been performed to exclude rare point mutations? Historically, in the German health care system even relatively low risks have served as an indication to offer genetic testing, as exemplified by the general offer of a prenatal chromosome analysis to all pregnant women from the age of 35. The risk of trisomy 21 among the offspring at this age is about 1:350 (0.29 %), rising to about 1:100 (1 %) at age 40 and to 1:23 (4.3 %) at age 45. Interestingly, while this procedure was widely accepted and routine practice internationally until it was replaced by NIPT in many countries, screening for other relatively common genetic risks in a preconceptional or prenatal setting has been a recurring subject of intense debate in many countries, including Germany.

## Aspects of genetic counseling

According to the German *Gene Diagnostics Law* (“Gendiagnostikgesetz”), carrier tests are predictive genetic tests and may therefore only be offered and initiated in the context of genetic counseling by board-certified medical geneticists or by physicians from other subspecialties with an additional qualification in genetic counseling [8]. Paragraph 9 of the law requires informing the patient about the “nature, significance and implications” of the genetic test. Infobox 1 summarizes some aspects about the use of a carrier test that are relevant, on the one hand, for the decision about a test offer by the physician and, on the other hand, for the decision of patients in whose family an autosomal recessive hereditary disease has occurred.


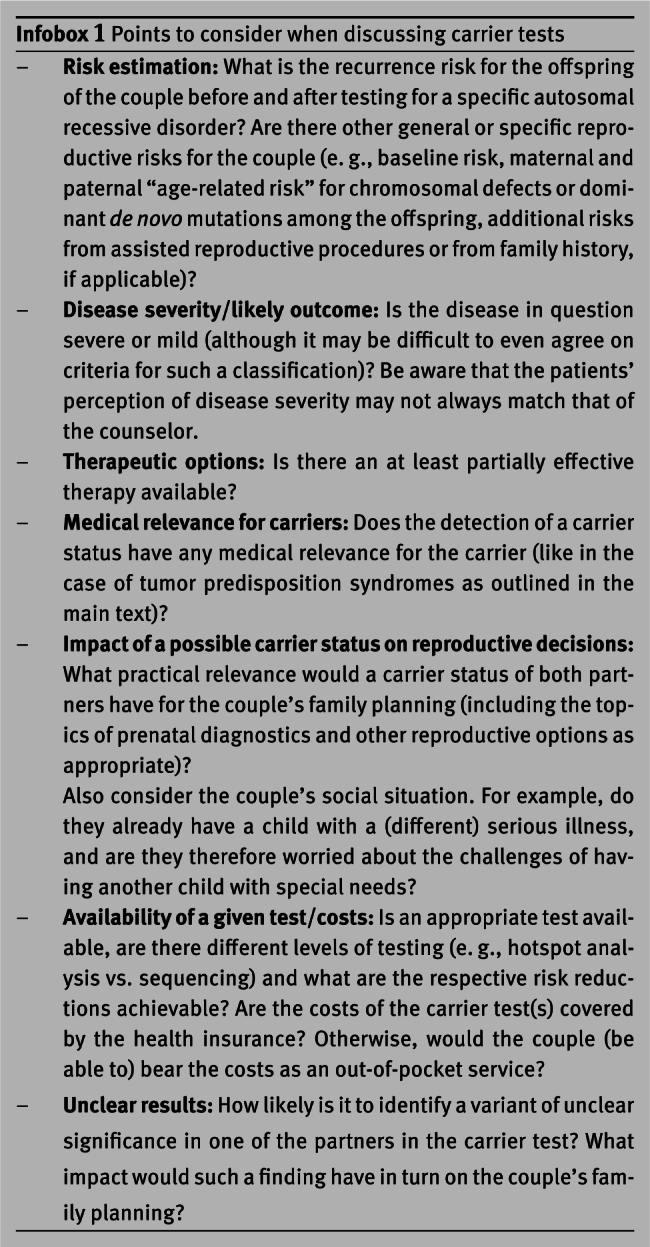


The last two points of Infobox 1 will be discussed in more detail below. Whether the reimbursement options for carrier tests for patients with statutory health insurance are currently sufficient is judged differently by the laboratories in Germany. In the case of private health insurances, reimbursement will often depend on the goodwill of the respective company and the subjective assessment of its medical assessors. If carrier tests are not covered by the health insurance, they could of course still be offered as out-of-pocket medical services. If such a practice were to prevail, it would mean that only financially well-off couples would be able to afford a comprehensive clarification of familial genetic risks, as required by the abovementioned guidelines – which we believe would raise pressing questions of justice and fairness. In addition to billing aspects, contract physicians of the statutory health insurance funds are also confronted with the question of when such diagnostics still fulfill the German socio-legal requirements of medical necessity (a notoriously fuzzy term [9]) and economic efficiency (a by no means less difficult term in this context). Clarifying a familial genetic risk can sometimes involve quite extensive genetic testing. Nevertheless, it may only reduce but not eliminate the risk for a genetic disease among the future offspring. This opens up a wide scope for interpretation and discussion.

Another relevant problem with carrier testing is the detection of variants of uncertain significance (VUS) according to the American College of Medical Genetics (ACMG) criteria for variant classification [10]. In combination with a clearly pathogenic mutation in the respective partner such variants imply a *possible* 25 % risk of having an affected child, but do not usually provide the couple with an option for prenatal or preimplantation genetic diagnosis. To our knowledge, it has not yet been systematically investigated how couples react to such a constellation of findings. There are also no databases in which information is available for genetic counseling on how likely the detection of a VUS in a gene is. In our experience, this probability is on average in the order of 1 %, but it can vary considerably from gene to gene. Thus, in many cases, it is significantly higher than the *a priori* probability of detecting a pathogenic variant in this gene (i. e., higher than the carrier rate of the respective disease in the relevant population). In practice, however, the detection of a VUS means that, instead of the hoped-for certainty, a couple in a vulnerable situation is left with additional uncertainty as a result of the carrier test. We therefore believe it is important to clearly articulate this point during genetic counseling. It may be possible to mitigate the problem by agreeing with patients not to report VUS in the context of carrier testing, as has been recommended for expanded carrier *screening* panels [11]. However, the ACMG criteria are very conservative and there are always cases, at least in our laboratory, where we are quite sure that we have identified a pathogenic variant but cannot (yet) classify it accordingly. Thus, such a strategy will sometimes only shift the dilemma and the responsibility for further action from the patient to the laboratory.

## Discussion and outlook

The discovery rate of genetic diseases is increasing rapidly due to technological progress, and thus the demand for carrier tests to clarify the recurrence risk of an autosomal recessive disease in a family will continue to increase. There is a lack of guidelines that offer direction as to which family constellations should serve as indication for an individual carrier test – and whether there are risks that are too low to be clarified. In the absence of such guidelines, it will often be left to chance whether couples interested in genetic diagnostics are offered a carrier test, or it will depend on their persistence. In the case of recurrence risks in the order of 1 %, most human geneticists in Germany will probably offer testing. For recurrence risks in the “per thousand” range, some will feel that such a risk may be negligible against the backdrop of the much higher baseline risk and may not even raise this point during genetic counseling. Others will fear possible legal liability in case such a low risk does eventually materialize, and a couple accuses them of having denied their request for this specific test, or of not excluding the risk to the absolute maximum of technical feasibility.

However, from an overarching perspective, such individual disease-specific carrier testing may not be a scalable testing strategy. It is well known that each individual is a carrier for several autosomal recessive diseases [12]. With the birth of an affected child, only one of these carrier states in the respective family branch becomes obvious for all family members – while people in whose family an autosomal recessive disease has never occurred may feel a false sense of security due to the lack of corresponding information and awareness. The distinction between the single carrier state recognized by family history and the many additional *unrecognized* carrier mutations seems arbitrary in this context, albeit psychologically understandable. Although the immediate trigger for the couple’s consultation may have been the carrier state for a single specific disease, the couple’s core concern is most likely that of genetic reproductive risks in general. Should this not, then, be fully addressed? Even relatively limited panels for *expanded carrier screening* (ECS) could cumulatively already clarify significantly higher reproductive risks of a couple than are present in most of the scenarios presented in Table 1. An ECS study of almost 350,000 individuals for 417 pathogenic variants in 94 genes mostly implicated in autosomal recessive disorders estimated – on the basis of the observed carrier frequencies – that the risk of having a child affected with one of these disorders (certainly representing a significant underestimate of overall risks) was 1:628 for Northern European couples and 1:275 for couples of African descent [12], [13]. Tests like these – either restricted to well-known pathogenic variants or screening entire genes for all (likely) pathogenic variants – are already offered in other countries and also by some laboratories in Germany. However, the cost of such carrier *screening* (as opposed to targeted carrier *testing*) would definitely have to be borne by the couples themselves at present. ECS also brings some important drawbacks compared to targeted testing: The gene panels may cover hundreds of genetic diseases that are not known in the family from personal experience – which represents a particular challenge for genetic counseling. The abovementioned problem of dealing with variants of unclear significance is also amplified, unless analyses are restricted to well-known pathogenic variants. But are these and other possible disadvantages [14] reason enough to ignore the availability of such tests in genetic counseling of couples concerned about their reproductive risks? Recommendations for the responsible implementation of expanded carrier screenings have been published by the *European Society of Human Genetics* in 2016 [15]. But proactively discussing ECS with couples seeking genetic advice is, in our experience, still not a widespread practice in Germany.

Being able to offer ECS on an individual basis to couples with concrete reproductive plans seeking to clarify their genetic risks as a part of genetic preconception care – and covered by health insurances – could be one way to address the abovementioned contradictory handling of reproductive risks. For Germany, this may not be realistic in the short term. Another way would involve formulating genetic preconception care guidelines, specifying the absolute risks above which family-specific carrier *testing* should be offered.

The examples we have described in this article will increasingly be encountered and dealt with in our everyday practice. Our discipline should therefore position itself with a common and unified strategy in this regard.
